# IQP-VV-102, a Novel Proprietary Composition for Weight Reduction: A Double-Blind Randomized Clinical Trial for Evaluation of Efficacy and Safety

**DOI:** 10.1155/2015/413075

**Published:** 2015-05-17

**Authors:** Barbara Grube, Udo Bongartz, Felix Alt

**Affiliations:** ^1^Practice for General Medicine, Kurfürstendamm 157/158, 10709 Berlin, Germany; ^2^Analyze & Realize GmbH, Weißenseer Weg 111, 10369 Berlin, Germany; ^3^Analyze & Realize GmbH, Waldseeweg 6, 13467 Berlin, Germany

## Abstract

The individual ingredients in IQP-VV-102 have demonstrated promising effects in reducing sugar and starch digestion, which potentially leads to weight loss. The trial objective was to evaluate the safety and efficacy of IQP-VV-102 in reducing body weight in overweight and obese subjects. 120 overweight and obese individuals aged 18 to 60 years were randomly assigned to 2 treatment arms (IQP-VV-102 and placebo). The trial was conducted in 2 study centres in Berlin, Germany. The primary efficacy analysis was conducted on 117 subjects (IQP-VV-102: *N* = 54; placebo: *N* = 59), comparing the weight loss effect at baseline and 12 weeks after randomization. There was a statistically significant reduction in mean body weight of 3.29 kg (SD 2.30) in the IQP-VV-102 group compared to 0.83 kg (SD 2.00) in the placebo group (*p* < 0.001). There were no serious or product-related adverse events that were reported over the combined period of 14 weeks. The findings suggested that IQP-VV-102 is effective and safe in body weight reduction in overweight and obese individuals in the short term. The study is registered under clinicaltrials.gov as NCT01681069.

## 1. Introduction

Overweight or obesity is a result of chronic imbalance between food consumption and energy expenditure. According to recently published data by WHO, up to 2008, an estimated 205 million men and 297 million women over the age of 20 are obese worldwide. The prevalence has almost doubled from 1980 to 2008 [1].

Obesity negatively impacts ones' health and its impact is shown across various body systems. Diseases associated with obesity range from cardiovascular diseases such as hypertension, coronary heart disease, and stroke to type 2 diabetes mellitus, osteoarthritis, and respiratory problems [2]. All these conditions could potentially lead to mortality and mandate an urgent need for safe and effective weight loss interventions. The reduction of dietary carbohydrate or sugar uptake by the body could be a potential approach to addressing obesity [3]. In an analysis of food consumption trends amongst obese individuals, dietary calorie increase was largely due to the increased intake of carbohydrates, especially refined carbohydrates [4, 5]. Despite the apparent health benefits of losing weight, more than 80% of obese individuals struggle to shed the pounds and also to maintain weight loss with the sole reliance on lifestyle and dietary modifications [6].

The investigational product, IQP-VV-102, is formulated with a proprietary blend of L- arabinose, a naturally occurring pentose, and grape marc extract. L-Arabinose works by inhibiting the hydrolysis of sucrose to glucose and fructose by intestinal sucrase, thus inhibiting glucose absorption in the intestine. Based on in vitro studies, L-arabinose selectively inhibits the intestinal sucrase activity in a noncompetitive manner [7, 8]. Through this inhibition, about 20% of the sucrase is maintained in the sucrase-L-arabinose complex for 6 hours [9, 10]. A study in mice fed sucrose and L-arabinose showed a reduced postprandial glucose and insulin response [7]. Another study in rats did not show any effect of L-arabinose on glucose response but reduced insulin concentrations were observed. L-Arabinose also prevented the increase of lipogenic enzymes activity and the increase in triacylglycerol concentrations in the liver [11]. Results from a clinical trial showed reduced postprandial glucose peak value and a reduction in the incremental area under the curve (iAUC) for insulin after supplementation with 4% L-arabinose in sucrose beverages [8].

Grape marc extract contains tannins that form complexes with essential carbohydrate digesting enzymes such as alpha-amylase and alpha-glucosidase. The inhibition of these enzymes was shown to reduce the breakdown of complex starch molecules and disaccharides, which in turn reduce carbohydrate digestion and absorption of calories derived from carbohydrates [10, 12]. In this study, we investigated the efficacy and safety of IQP-VV-102 in a randomized controlled trial to test the hypothesis that dietary supplementation with L-arabinose and grape marc extract promotes greater weight loss compared to placebo in overweight and moderately obese individuals.

## 2. Material and Methods

### 2.1. Study Participants

Eligible subjects included obese and overweight (25 kg/m^2^ ≤ BMI ≤ 35 kg/m^2^) Caucasian males and females, aged between 18 and 60 years old, with expressed interest to lose weight. The subjects were also accustomed to 2 to 3 main meals a day with a consistent and stable body weight for 3 months prior to study enrolment. Other weight loss products or programs were prohibited during the study duration. Women of childbearing potential had agreed to use appropriate birth control method during the entire trial period. Subjects with known sensitivity to any source of the active ingredients and excipients were excluded from the study.

Other exclusion criteria included presence of any endocrine disorders, uncontrolled thyroid dysfunctions, history of eating disorder, usage of medication which could influence body weight within 3 months prior to enrolment, unstable hypertension, history of gastrointestinal, psychiatric, or any serious diseases that could influence the outcome of the study, history of bariatric or abdominal surgery, intake of antiobesity drugs, use of medication that affects GI function within 3 months prior to enrolment, participation in strenuous sports activities (>3 hours per week), alcohol or drugs abuse, smoking cessation in the previous 6 months, inability to comply with study requirements, participation in other studies 30 days before enrolment, excursion of clinical safety parameters, and pregnant and lactating women.

All subjects voluntarily gave written informed consent. The clinical investigation was approved by the Ethics Committee of the Ethikkommission der Charité Universitätsmedizin Berlin and was performed in compliance with the principles of the World Medical Association (Declaration of Helsinki), the EU recommendations for Good Clinical Practice (CPMP/ICH/135/95), ICH E6 (R1), and EN ISO 14155:2011.

### 2.2. Experimental Design and Intervention

This double-blind, randomized, placebo-controlled, parallel group study was conducted at 2 study centres in Germany from November 2012 to June 2013.

The study was conducted over 14 weeks, including a 2-week placebo run-in phase and a 12-week treatment phase. Subjects who achieved treatment compliance of 80% to 120% and dietary compliance (body weight reduction of −0.1 to 4 kg and deviation of less than 20% from the prescribed daily calorie intake) during run-in were randomized in a 1 : 1 ratio to either the IQP-VV-102 group or placebo group.

Throughout the 12-week treatment period, subjects took either 2 tablets of IQP-VV-102 (600 mg L-arabinose and 45 mg grape marc extract per tablet) or a matching placebo, 2 times a day, before the two heaviest meals of the day. The placebo tablet was physically identical to IQP-VV-102 and contained only the inert excipients of cellulose, tricalcium phosphate, and magnesium stearate.

Randomization was done using a block size of 4 by an independent biostatistician. The assignment of the random numbers to both the IQP-VV-102 and the placebo was performed externally (by an independent pharmacist) and prior to the start of the study.

All subjects were instructed to maintain a nutritionally balanced, mildly hypocaloric diet, with 55% of energy from carbohydrates. The daily energy requirement was estimated (for each subject) according to the Institute of Medicine's equations for estimating energy requirements [13], based on gender, age, physical activity rate, and actual body weight. In order to induce weight loss regardless of treatment received, the daily energy requirement was reduced by 20% from the calculated value, according to clinical investigation plan. Modular diet plans for 5 levels of energy (1500, 1800, 2000, 2200, and 2500 kcal/day) were provided to all subjects, which included at least 30 daily diet plans. In addition, subjects received a brochure covering the 1000 most common food items with information on energy and fat contents. This was to enable the subjects to adhere to the energy level they were assigned to.

Subjects were also encouraged to gradually increase physical activities of moderate intensity such as walking or cycling. Diet plans and diaries were distributed to subjects during visit 1, together with the usage instructions. During all subsequent visits, the investigators reviewed subjects' adherence to diet plans and physical activity. The assessment was based on diaries, which recorded food intake and standardized weekly physical activity.

### 2.3. Measurements

The primary efficacy parameter is difference in mean loss of body weight (kg) between the second visit (baseline, after the 2-week run-in phase) and fifth visit (end of study, after 12-week treatment). Measurement of body weight was done using calibrated weighing scales (TanitaBC-420 SMA). The same scales also measured the subjects' body fat content using bioelectrical impedance analysis. These measurements were taken during baseline, week 5, and week 12 in clinic by the investigators.

Secondary efficacy parameters were proportion of subjects who lost at least 3% and 5% of baseline body weight, changes in waist circumference, changes in hip circumference, changes in waist-hip ratio, changes in body fat mass (% and kg), and fat free mass (kg). These parameters were measured at randomization, week 4, week 8, and week 12 during the treatment period.

The investigators and subjects rated the efficacy of the product at week 12 using a questionnaire.

Waist circumference (in cm) was determined using a measuring tape to ascertain the distance between the lateral lower rib midway margin and the iliac crest. The maximum circumference of the hip was measured as hip circumference (in cm). Safety assessments comprised blood tests (carried out at screening visit and after 12 weeks of intervention) which evaluated hematology, liver function parameters (alanine transaminase, aspartate aminotransferase, gamma-GT, alkaline phosphatase, and bilirubin), renal function parameters (creatinine, urea), lipid metabolism parameters (triglycerides, LDL- and HDL-cholesterol, and total cholesterol), protein metabolism parameter (uric acid), and carbohydrate metabolism parameters (fasting blood glucose, HbA1c). All blood samples were analyzed centrally (Medizinisch-Diagnostische Institute, Berlin, Germany). Subjects' blood pressure was measured during each visit. All adverse events that occurred were documented. Both investigators and subjects evaluated the safety and efficacy of the investigational product based on their personal opinion on a questionnaire.

### 2.4. Statistical Analysis

All data were analysed using the SPSS Statistic software, version 19.0 (SPSS, Chicago, IL).

The sample size was determined through effect size. The standardized mean difference between IQP-VV-102 and placebo group was measured and subsequently standardized to the distribution of the observed value. The minimum sample size of 120 subjects was required, factoring in a dropout rate of 20%. Using the Mann-Whitney *U* test, the power was adjusted to 80%, with a significance level of 5% (two-tailed test). There were limited experiences with IQP-VV-102; hence, an effect size of 0.6 was assumed in conformity with several published studies.

Primary and secondary endpoints and safety variables were measured through descriptive statistics. For continuous data, the number, mean, standard deviation, median, extremes, and quartiles were measured at the specified time points. For ordinal data, frequency of distribution was analysed. All categorical data were summarized using frequency tables. The values of metric data can be merged in ordinal classes according to clinical criteria to determine their frequency distribution.

The study was carried out in a double-blind design, with an identical placebo—neither the investigator nor the subject will be aware of the treatment assigned. Bias in treatment allocation was eliminated by blocks of 4 randomizations.

Analyses were performed on the intent-to-treat (ITT) population. Per protocol (PP) population was also analysed for primary endpoints.

## 3. Results

**Figure 1:**
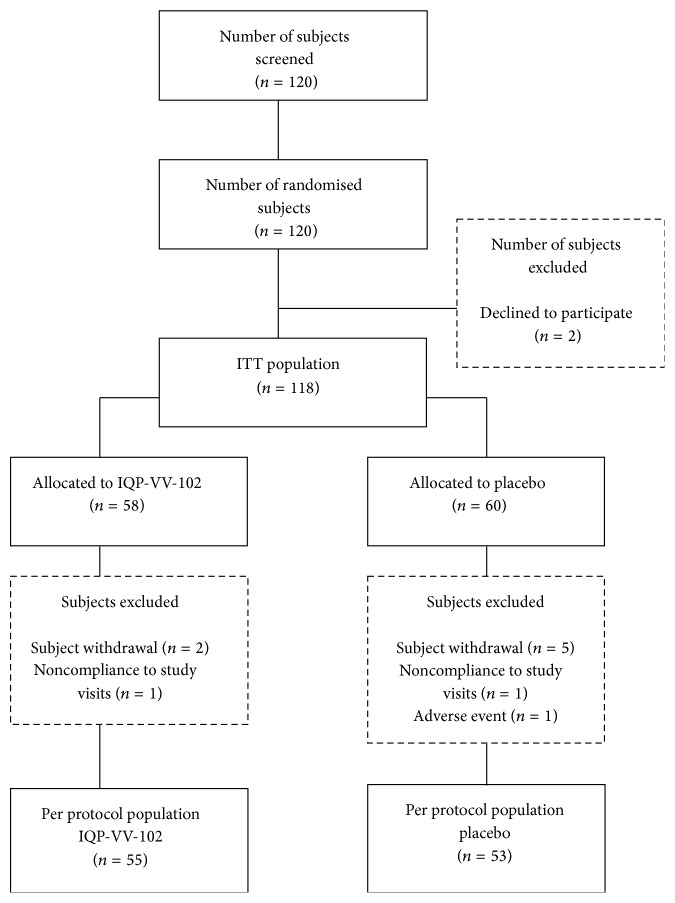
Study design and disposition of subjects.

A total of 120 subjects were recruited into the study from November 2012 to February 2013. All subjects complied with the treatment regimen during the run-in phase and were randomized into the study. 118 subjects were included in the intention-to-treat (ITT) population, which consisted of 58 subjects from the IQP-VV-102 group and 60 subjects from the placebo group. Two subjects were excluded due to voluntary study withdrawal (Figure 1). A total of 117 subjects from the ITT population were included in the efficacy analysis because one subject was lost to follow-up after visit 3.

Subjects recruited in the study were Caucasians, with the mean age of 42.0 (SD 11.8) years, and 71.2% of subjects were female. Similar baseline and demographic characteristics were observed in both groups (Tables 1 and 2).

### 3.1. Efficacy

#### 3.1.1. Body Weight

**Figure 2:**
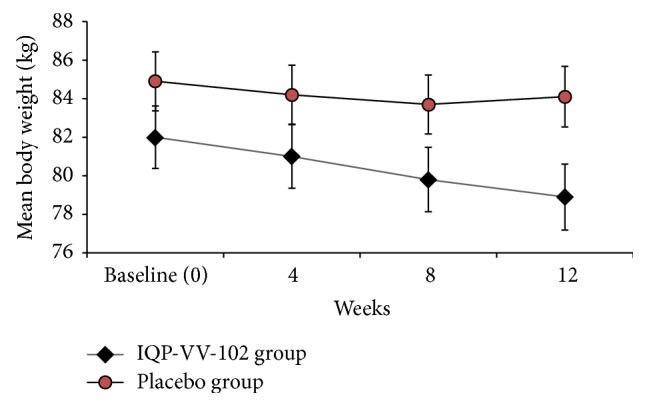
Body weight reduction over time (error bars represent SEM).

Figure 2 shows the trend in body weight reduction over 12 weeks. The mean body weight of the subjects at baseline (V2) was 82.0 kg (SD 10.6) in the IQP-VV-102 group and 84.9 kg (SD 13.0) in the placebo group, which showed no significant difference (*p* > 0.05). After 12 weeks (V5), subjects on IQP-VV-102 lost significantly more weight than subjects on placebo. The IQP-VV-102 group lost a mean of 3.29 kg (SD 2.30), while the placebo group lost a mean of 0.83 kg (SD 2.00), with an overall difference of 2.46 kg (*p* < 0.001). The difference in weight change between the two groups was significant as early as week 8 (V4) (mean loss of 2.17 kg in the IQP-VV-102 group versus mean loss of 1.23 kg in the placebo group, *p* < 0.001).

A subgroup analysis of mean body weight reduction based on subject's baseline BMI was conducted. After 12 weeks, the IQP-VV-102 treated group exhibited significantly more body weight reduction of 3.18 kg (SD 3.12) compared to the 1.88 kg (SD 2.04) in the placebo treated group (*p* = 0.012) in the obese subgroup (BMI ≥ 30 kg/m² at V2). Meanwhile in the subgroup of overweight subjects (BMI < 30 kg/m² at V2), the IQP-VV-102 group exhibited statistically significant body weight reduction of 3.35 kg (SD 1.76) compared to the 0.42 kg (SD 1.85) in the placebo group (*p* < 0.001).

A total of 77.2% of subjects achieved more than 3% weight reduction at week 12 (V5) in the IQP-VV-102 group as compared to 13.3% in the placebo group. In the IQP-VV-102 group, 40.4% of the subjects achieved 5% or more weight reduction at week 12 (V5), compared to 3.3% in the placebo group.

In another subgroup analysis on body weight reduction based on gender in the IQP-VV-102 group, male subjects lost a mean weight of 3.68 kg compared to placebo 1.00 kg (*p* < 0.001) while female subjects lost a mean weight of 3.2 kg compared to placebo 0.73 kg (*p* < 0.001).

#### 3.1.2. Waist Circumference

The mean waist circumference of subjects in the IQP-VV-102 group was 99.8 cm (SD 8.7) while the placebo group was 99.1 cm (SD 8.9) (*p* > 0.05). Subjects supplemented with IQP-VV-102 lost a mean of 3.17 cm in waist circumference while subjects in the placebo group lost a mean of 0.61 cm after 12 weeks (V5), with an overall difference of 2.56 cm. The difference between the two groups is statistically significant (*p* < 0.001).

#### 3.1.3. Hip Circumference

The mean hip circumference in the IQP-VV-102 group at baseline (V2) was 108.1 cm (SD 6.7)  while the placebo group was 1.30 cm (SD 2.22) (*p* < 0.001). After 12 weeks (V5), subjects in the IQP-VV-102 group experienced a significantly higher reduction in hip circumference compared to subjects in the placebo group (mean reduction of 2.99 cm (2.04)  in the IQP-VV-102 group and 1.30 cm (SD 2.22) in the placebo group).

#### 3.1.4. Body Fat Mass (kg)

After 12 weeks (V5), there was a statistically significant mean reduction of 2.14 kg in body fat mass in the IQP-VV-102 group, compared to a mean decrease of 0.56 kg in the placebo group; with an overall difference of 1.58 kg (*p* = 0.001). In the subgroup analysis of overweight subjects, the IQP-VV-102 group exhibited statistically significant body fat mass reduction of 2.57 kg (SD 2.32) compared to the 0.38 kg (SD 2.31) in the placebo group (*p* < 0.001). The subgroup analysis of obese subjects indicated no statistically significant difference between the IQP-VV-102 group and placebo group.

#### 3.1.5. Body Fat Content (%)

After 12 weeks, there was a statistically significant mean reduction of 1.52% in body fat mass in the IQP-VV-102 group, compared to a mean decrease of 0.22% in the placebo group; with an overall difference of 1.3% (*p* = 0.024).

### 3.2. Safety and Tolerability

No statistically significant changes were observed between baseline and 12 weeks in mean blood pressure and mean heart rate. Analysis of blood profiles and clinical chemistry did not reveal any clinically significant changes throughout the study. At the end of the study, subjects in the IQP-VV-102 group rated the safety of IQP-VV-102 as “very good” or “good” in 100% of cases, whilst the placebo was rated as “very good” or “good” in 98.2% of cases (*p* = 0.046).

During the study, 16 adverse events were documented in 13 subjects. AEs included bruised left thigh, suspected new case of hypothyroidism, migraine, postmenopausal complaints, urinary tract infection, sore throat, fever, tonsillitis, acute gastroenteritis, headaches (repeated/single episode), diarrhoea, acute tonsillitis, and common cold. All adverse events were not related to the investigational product and were not serious or severe.

## 4. Discussion

The animal and human studies for each individual ingredient in IQP-VV-102 showed promising results in the reduction of dietary carbohydrate absorption and it was thus hypothesized that this could eventually lead to a weight reduction effect. To date, no clinical study has been conducted to demonstrate the weight loss effect of the combination of grape marc extract and L-arabinose. The present study is intended to evaluate the safety and the potential of IQP-VV-102 to promote body weight loss in a randomized, double-blind, placebo-controlled, parallel study involving Caucasian subjects.

IQP-VV-102 has demonstrated superior body weight reduction compared to placebo as early as 8 weeks of consumption in this study. At the end of the 12 weeks of study, subjects who consumed IQP-VV-102 lost significantly more body weight in comparison to subjects who were on placebo (3.29 kg (SD 2.30) versus 0.83 kg (SD 2.00), *p* < 0.001). 40.4% of subjects lost more than 5% body weight at week 12 (V5) in the IQP-VV-102 group as compared to 3.3% in the placebo group, which is associated with reduced risk of metabolic syndrome defined according to the criteria from the National Cholesterol Education Program's Adult Treatment Panel III. [14].

Statistically significant waist and hip circumference reduction were also observed in subjects after consumption of IQP-VV-102 for 8 weeks. The reduction of waist circumference, which is a key indicator of central obesity, is linked to the risk reduction of cardiovascular diseases, dyslipidemia, and diabetes mellitus [15, 16]. The body fat reduction of subjects on IQP-VV-102 was also superior compared to that of the placebo group. This was especially apparent in overweight subjects.

The study was limited by a 12-week treatment period, and no follow-ups were included to observe the weight maintenance effect of IQP-VV-102. Rebound weight gain was observed in longer term studies [17, 18], and future investigation is needed to explore the long term weight loss and weight maintenance effect of IQP-VV-102.

Another study limitation was the self-reporting of energy intake. Despite the effort of investigators to ensure subject diet compliance, previous data indicated that self-reported food intake among the obese had high variance [19, 20].

There was a high compliance rate to the IP consumption (>99%). This indicates that IQP-VV-102 dosing regimen (2 tablets twice a day) is generally well tolerated and optimum weight loss is achievable under real-life conditions. There were no serious or product-related adverse events reported and no clinically significant changes in the safety parameters.

## 5. Conclusion

IQP-VV-102 is efficacious for body weight management in conjunction with good dietary and exercise habits. Good safety and tolerability have also been demonstrated in overweight and obese but otherwise healthy subjects.

## Figures and Tables

**Table 1:** Demographic characteristics in intention-to-treat population.

Parameter	Intention-to-treat population (*n* = 118)		*p* value
	IQP-VV-102 group (*n* = 58) mean (SD)	Placebo group (*n* = 60) mean (SD)	
Gender			
Males	12 (20.7%)	22 (36.7%)	0.055
Females	46 (79.3%)	38 (63.3%)	

Age (years)	43.6 (11.6)	40.5 (11.9)	0.163

**Table 2:** Mean changes in primary and secondary parameters between baseline and week 12.

Parameters	IQP-VV-102 group (*n* = 57)	Placebo group (*n* = 60)	*p* value
	Mean change (SD)	Mean change (SD)	
Body weight (kg)	3.29 (2.30)	0.83 (2.00)	<0.001
Waist circumference (cm)	3.17 (2.55)	0.61 (2.23)	<0.001
Hip circumference (cm)	2.99 (2.04)	1.30 (2.22)	<0.001
Body fat content (kg)	2.14 (3.42)	0.56 (2.43)	0.001
Body fat content (%)	1.52 (3.85)	0.22 (2.46)	0.024
