# Corrigendum to “IQP-VV-102, a Novel Proprietary Composition for Weight Reduction: A Double-Blind Randomized Clinical Trial for Evaluation of Efficacy and Safety”

**DOI:** 10.1155/2016/7173896

**Published:** 2016-12-29

**Authors:** Barbara Grube, Udo Bongartz, Felix Alt

**Affiliations:** ^1^Practice for General Medicine, Kurfürstendamm 157/158, 10709 Berlin, Germany; ^2^Analyze & Realize GmbH, Weißenseer Weg 111, 10369 Berlin, Germany; ^3^Analyze & Realize GmbH, Waldseeweg 6, 13467 Berlin, Germany

In the article titled “IQP-VV-102, a Novel Proprietary Composition for Weight Reduction: A Double-Blind Randomized Clinical Trial for Evaluation of Efficacy and Safety” [1], there was an error under Section 3.1.3: Hip Circumference, which should be corrected as follows.

*3.1.3. Hip Circumference*. The mean hip circumference in the IQP-VV-102 group at baseline (V2) was 108.1 cm (SD 6.7) while the placebo group was 106.5 cm (SD 6.5) (*p* = 0.101).

In addition, the declaration statement in the Conflict of Interests section “The authors declare that there is no conflict of interests regarding the publication of this paper” should be replaced with the following declaration “InQpharm Europe Ltd. is the manufacturer of the investigational product and oversaw the trial and manuscript preparation as the sponsor.”
